# Comparing student, instructor, and expert perceptions of learner-centeredness in post-secondary biology classrooms

**DOI:** 10.1371/journal.pone.0200524

**Published:** 2018-07-11

**Authors:** Ashley B. Heim, Emily A. Holt

**Affiliations:** School of Biological Sciences, University of Northern Colorado, Greeley, CO, United States of America; University of Westminster, UNITED KINGDOM

## Abstract

Learner-centered classrooms encourage critical thinking and communication among students and between students and their instructor, and engage students as active learners rather than passive participants. However, students, faculty, and experts often have distinct definitions of learner-centeredness, and the paucity of research comparing perspectives of these different groups must be resolved. In the current study, our central research question was *how do student*, *faculty*, *and expert observer perceptions of learner-centeredness within biology classrooms compare to one another*? We sampled 1114 students from fifteen sections of a general biology course for non-majors, and complete responses from 490 students were analyzed. Five valid and reliable tools (two faculty; two student; and one expert observer) evaluated the learner-centeredness of each participating section. Perceptions of learner-centered instructors often aligned with those of expert observers, while student perceptions tended not to align with either group. Interestingly, students perceived learner-centered instructors as less learner-centered if they taught at non-traditional times and/or in large-enrollment sections, despite their focus on student learning. Perceptions of learner-centeredness in the biology classroom are complex and may be best captured with more than one instrument. Our findings encourage instructors to be cognizant that the approaches they employ in the classroom may not be interpreted as learner-centered, in the same manner, by students and external observers, particularly when additional course factors such as enrollment and scheduling may encourage negative perceptions of learner-centered practices.

## Introduction

Active learning is broadly defined as engaged teaching approaches that encourage critical thinking and communication among students and between students and their instructor [1–3]. Further, active learning contributes to the learner-centeredness of a classroom, which can also be characterized by the level of bilateral learning in a course, and whether students have a role in this process as active learners rather than passive participants [4]. While active classrooms tend to share goals of higher cognitive learning and separate the roles of instructors and students in a similar way, they can, on the ground, look very different, depending on the learner-centered practices administered in the classroom.

Experts within education fields have developed these broad descriptions of learner-centeredness and learner-centered practices. However, as Andrews et al. [5] noted, the definition of a “learner-centered” classroom is often generated by the instructors or students themselves, generally documented through self-reported survey responses in educational research. It remains unclear to what degree these expert, instructor, and student definitions of learner-centeredness can be interwoven or if they are discrete, potentially diverging perceptions.

### Student challenges with learner-centered classrooms

Learner-centered classrooms reportedly lead to improvements in students’ metacognitive abilities, critical thinking skills, and subject knowledge [6–14], and have also been linked with improvements in student performance in the classroom [1, 12–13, 15]. Further, increases in student motivation, persistence, self-confidence, and attitudes in science fields have been correlated with learner-centered teaching and learning approaches in STEM (i.e., science, technology, engineering, and technology) courses [16–18]. The multi-faceted, positive impact on students from active learning [6, 19] is of particular significance in light of the continued leakiness of the STEM pipeline [20–21]; perhaps by actively engaging students in STEM courses from the start of their undergraduate careers, instructors can both increase retention rates and ensure a more authentic experience in the sciences for incoming students.

Despite these numerous benefits, many students resist learner-centered pedagogies. University students often have mixed feelings about the use of active learning techniques in lecture [15, 17]; several studies have reported that students prefer traditional lectures over active learning and consider the former method of teaching more conducive to learning [22–24]. Herreid and Schiller [25] noted that students often feel more learner-centered classrooms (i.e. the flipped classroom) require more out-of-class time for reading, homework, etc., than traditional classrooms. Clicker questions or small group discussions in lectures, which require self-directed learning and critical thinking of students, have been shown to leave some students feeling frustrated or withdrawn from the course [26]. Similarly, Cooper and Brownell [27] reported that students of the LGBTQIA community often feel unwelcomed in active learning biology lectures and perceive increased pressure to reveal their identities during the frequent group learning activities characteristic of such sessions. While their study focused on a particular population of students, arguably the transition to a more active classroom likely increases scholastic accountability and social pressure on all students as they are forced into a more collaborative learning environment.

In a study by Watters & Watters [28], first-year undergraduate biochemistry students reported that they believe effective learning involves information transfer and prefer surface to deep strategies. Therefore, if students understand “learner-centered teaching” as strategies which maximize student learning, which they may erroneously equate with lecture-style presentations, their interpretations of learner-centeredness in the science classroom may be quite skewed from those of instructors and experts. Tsang and Harris [24], who found that students are unfamiliar with pedagogical practices and the process of learning in general, supports the presence of these student misconceptions. Subsequently, students’ negative perceptions of truly learner-centered classrooms and their unwillingness to engage in these practices may be rooted in their misconception that the extra expectations are burdens rather than benefits to them [29].

### Faculty challenges with learner-centered classrooms

As mentioned above, learner-centered practices may improve student-faculty relations [18], which consequently improve the overall quality of the classroom environment by providing increased opportunity for discussion amongst the class [30] and shifting the accountability and responsibility of learning from the instructor onto the student [29]. Despite these reported benefits, many instructors remain hesitant to translate learner-centered pedagogies into their current teaching practices, citing lack of support and training [17, 31–32], increased time and effort required to reform a class [17, 24, 33], and loss of “professional identity” [34]. Some instructors view the lab component of a course as sufficient engagement and thus fail to incorporate active learning approaches in lecture, demonstrating a form of passive resistance [16, 35]. Andrews et al. [5] argues that the link between active learning and increased student learning gains may be attributed to instructors’ pedagogical experience and not the teaching strategy itself. These findings combined with personal ambivalence may deter science faculty from reforming their classrooms, which helps to explain the persistence of didactic lecture [11] in the face of contradictory evidence.

However, a gradual shift from traditional lecturing to more active strategies is occurring in undergraduate courses [36], and individual instructors are reforming their classes and experimenting with more learner-centered strategies. Regretfully, approximately 75% of instructors that Ebert-May et al. [37] surveyed claimed that they used learner-centered practices but in fact used a lecture-based, teacher-driven pedagogy, demonstrating a large disconnect between faculty perceptions and actual teaching practices. This disconnect may derive from the possibility that instructors have their own disparate definition of learner-centeredness compared to students and expert observers, or perhaps because instructors undergo a cognitive shift after pedagogical development that is not necessarily transferred to their actual classroom practices [38–39]. Dall’Alba and Sandberg [40] note that, even after educators complete professional development programs, a broad understanding of pedagogical practice is uncommon among participants; the authors further argue that professional development not only incorporates development of skills but knowledge and attitudes as well, which could at least partially explain the aforementioned disconnect between instructors’ perceptions of learner-centeredness compared to those of experts. Further, McCombs and Quiat [41] found that student perceptions tended to be a better measure of learner-centeredness than instructor perceptions and that, additionally, these student perceptions were more aligned with those of trained educational and developmental psychologists rather than the perceptions of course instructors [42].

### Instruments for measuring learner-centeredness

A variety of valid and reliable instruments are available to analyze the learner-centeredness of a classroom, whether from the perspective of the student, the instructor, or an expert observer. Previous work has used some of these tools to contrast why students learn and how they learn [43–46], and how the teaching-learning environment influences student approaches to studying and learning [47–48]. Faculty instruments provide teachers formal opportunities for self-reflection and -assessment. Data from these tools may serve as a compass to focus reform efforts to best achieve a student-driven learning environment [49–51]. Meanwhile, expert observer protocols are often used to enhance student learning via critiquing and reforming teaching practices from an objective vantage point. Such protocols can quantify the learner-centeredness of instruction in a classroom, providing meaningful feedback to the instructor [52–54].

Many previous studies measure the degree of learner-centeredness of classrooms from just a single perspective: only the student view [43–48], only the instructor view [49–51], or only the expert view [52–54], based on a single instrument; yet, there is a dearth of studies which cross-evaluate student, faculty, and expert perceptions. As students, faculty, and experts often have distinct definitions of learner-centeredness, the paucity of research based on instruments which capture the perspectives of these different groups must be resolved. One exception, Trigwell et al. [55], compared faculty and student perceptions with separate faculty (i.e. the Approaches to Teaching Inventory, or ATI) and student tools (i.e. the Study Process Questionnaire, or SPQ). They found student and faculty perspectives on learner-centeredness generally agreed [55]. In courses where instructors self-reported a more teacher-centered focus on transmitting knowledge, students adopted a more surface approach to learning that subject; in contrast, but less strongly, in courses where instructors self-reported a more student-centered focus on conceptual change, students adopted a deeper approach to learning [55]. These findings were not compared to an expert observer’s perceptions of learner-centeredness and therefore may have incorporated bias due to instructors’ over-estimation of teaching skills or students’ resistance or lack of pedagogical knowledge regarding learner-centeredness.

In another study, Gibbs and Coffey [56] compared an instructor tool to two student surveys and found that instructors, who were pedagogically trained, tended to believe that they were encouraging deeper learning approaches compared to instructors who received no pedagogical training. While student learning gains improved in courses with pedagogically trained versus untrained instructors, student scores on the “Deep Approach” subscale of a student questionnaire did not significantly increase; in contrast, student learning gains remained unchanged in courses taught by the untrained cohort of instructors [56]. This study suggests that students may be misjudging their learning by performing at a high level but not attributing that success to learner-centered approaches; meanwhile, instructors of their sample who participated in pedagogical training appear more likely to use learner-centered teaching practices and may excel in such aspects of teaching as enthusiasm, organization, and rapport [56].

The current study is unique in that it used several student and instructor instruments from each perspective within the same classroom, and compared these perspectives to one another in addition to expert perceptions of the same biology classrooms. Redundancy in tools for individual populations can allow us to capture different elements of learner-centeredness, providing a more complete understanding of how learner-centeredness is perceived in the undergraduate biology classroom.

### Purpose and research questions

In the current study, our central research question was *how do student*, *faculty*, *and expert observer perceptions of learner-centeredness within biology classrooms compare to one another*? Specifically, we wanted to (a) compare subscales within individual student and faculty instruments, (b) compare subscales across student, faculty, and expert observer instruments and describe those relationships, and (c) describe the structure of learner-centered classrooms using multiple instruments. We predicted that different instruments, or subscales within a single instrument, measuring learner-centeredness from a single perspective (i.e., faculty or student) would both linearly and positively correlate. We envisaged that faculty perceptions would generally be disconnected from expert perceptions, as supported by Ebert-May et al. [37]. Contrastingly, we predicted that student perceptions would be more aligned with expert perceptions, as supported by McCombs and Quiat [41] and Daniels et al. [42]. We also predicted that student perceptions of learner-centeredness would be disconnected from faculty perceptions, supported by Fraser’s [57] findings that student perceptions of instruction and the overall class environment are more negative than instructor perceptions, even in post-secondary education. We hypothesized that a single-dimension framework, characterized by highly learner-centered at one end and highly teacher-centered at the opposing end, would best describe biology classrooms from various perspectives.

## Materials and methods

### Ethics statement

The procedures for this study were approved by the Institutional Review Boards of Utah Valley University (IRB# 01103) and the University of Northern Colorado (IRB #932641–1). Written informed consent was obtained by all participating students and faculty at the beginning of the study.

### Participants

We conducted an observational study in introductory biology classrooms at one public post-secondary institution in the western US. While this institution is self-described as “engaged” in its mission, instructors were not considered pedagogical experts. We assumed that the fifteen class sections and nine instructors in our study were representative of average undergraduate biology classrooms.

We sampled 1114 students from fifteen sections of a general biology course for non-majors, and complete responses from 490 students were analyzed (i.e., students who completed both the student surveys administered in this study). While volunteer participation can result in non-response bias, our response rate of 44% is proximal to the accepted average noted in psychological studies [58] when considering the removal of three course sections from the original data set (n = 244 students enrolled; further described below). Our twelve participating class sections varied by student enrollment (min = 16 students per section, max = 391, mean = 91.4) and class meeting time (1 section was a weekend course, 3 were night classes, and 8 met during the weekday).

Nine instructors taught these fifteen sections during Fall 2013 and Spring 2014; six of these instructors taught two sections during the same semester. One of the participating instructors failed to complete both faculty surveys, and consequentially both of this instructor’s sections were removed from our data set (n = 94 students enrolled). Additionally, one of the participating instructors voiced concern after completing the faculty surveys regarding their inconsistent interpretation of survey questions; to prevent a lack of validity and reliability in our analyses, we also removed this instructor’s section from our data set (n = 150 students enrolled). Our final analyses included twelve sections. The remaining seven instructors had various levels of teaching experience: one instructor had taught for 2–3 years; one for 3–5 years; two for 11–20 years; and three for 21 or more years. Additionally, the population of instructors used in this study included tenured and tenure-track professors, as well as adjunct instructors. Course section numbers used in this paper (1–12) reflect their ranked Reformed Teaching Observation Protocol (RTOP) score (i.e., section one had the highest RTOP score, while section twelve had the lowest RTOP score), and to protect participant anonymity do not link to actual institutional numbering schemes.

### Conceptual framework

**Fig 1:**
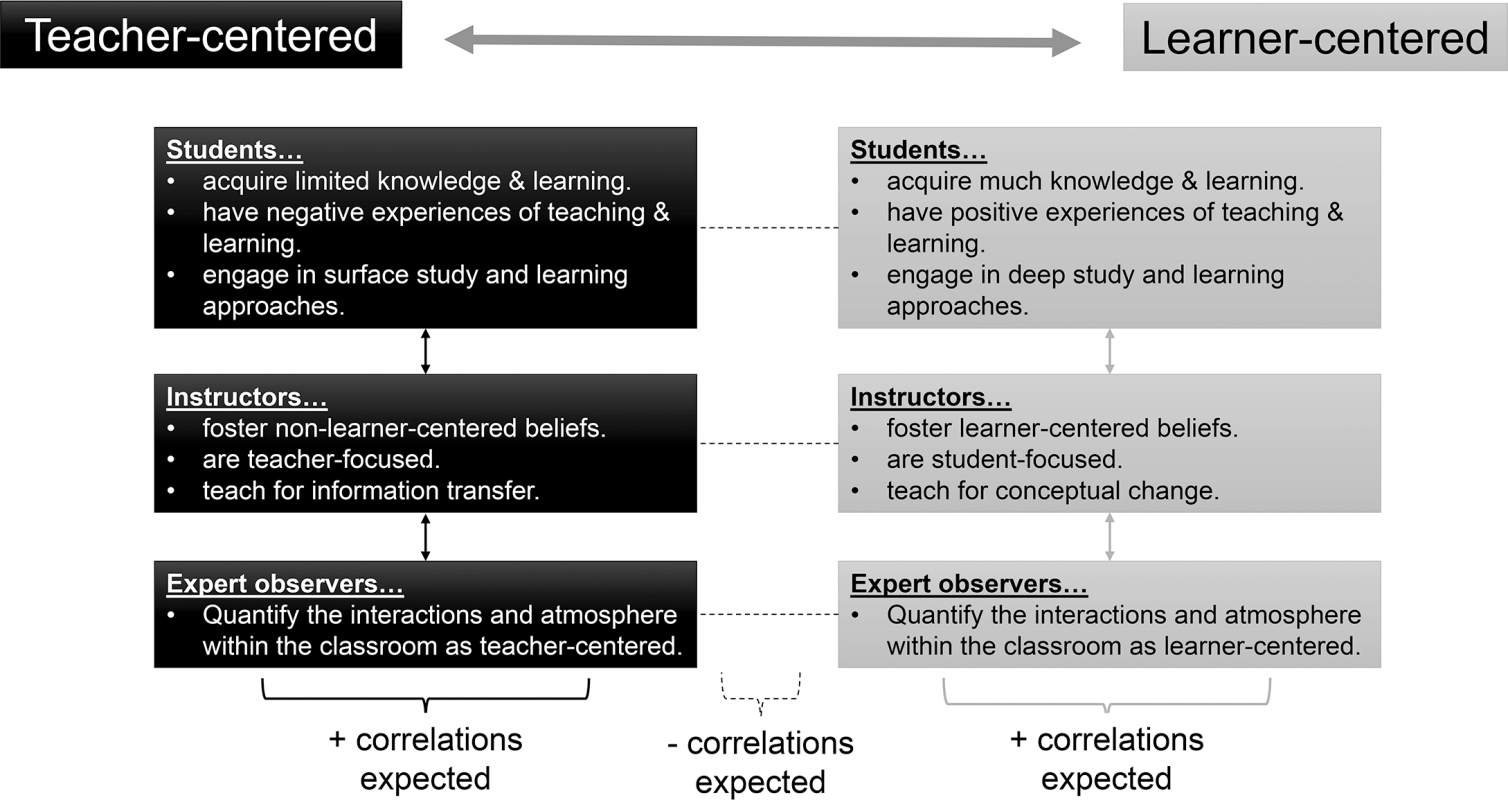
The proposed one-dimensional learner- to teacher-centered framework. Examples of student behaviors and instructor practices at the learner-centered end (in gray) juxtapose those that are more teacher-centered (black) at the other end of the framework. Learner-centered descriptors (gray) were expected to positively correlate with each other, while teacher-centered descriptors (black) were expected to positively correlate with each other. Negative correlations (dashed line) were expected between two related but contrasting descriptors, as both would fall on opposite ends of the learner- to teacher-centered framework. For example, deep approaches are more learner-centered, while surface approaches are more teacher-centered; a student that engaged in deeper learning approaches would not be expected to engage in as many surface approaches, or vice versa.

We used five valid and reliable tools (2 for faculty, 2 for students, and 1 for expert observers) to evaluate the learner-centeredness of each section participating in this study. The conceptual framework, or null hypothesis, for our work is a one-dimensional gradient, where a tool or subscale within an instrument falls at either end of a learner- to teacher-centered gradient, concomitantly opposing the other end (Fig 1). We expect the student-centered end of our gradient to include classrooms where faculty hold more learner-centered beliefs and focus more on conceptual change in their students, and where students incorporate deeper learning approaches and dedicate more class time to building models and sharing ideas with one another. In contrast, at the opposing end of our gradient, we expect a more teacher-centered classroom to include more non-learner-centered beliefs and be more focused on information transfer by faculty to students, and for students to incorporate more surface learning approaches and rarely interact with the instructor or their peers during class.

We assumed that subscales or factors of different instruments would overlay onto our conceptual framework (Fig 1), and likewise relate to other tools positioned within this framework. If factors, from different instruments or within the same instrument, both attempted to capture learner-centered behaviors, we expected that those factors would positively covary, and fall at the same end of our gradient. Alternatively, we predicted that if one subscale measures teacher-centered beliefs and another measures learner-centered beliefs, they will negatively covary, representing opposite ends of our 1-D framework.

### Instruments for comparing perceptions of learner-centeredness

Nine factors (*italicized*) were derived from five published instruments (Table 1) to describe learner-centered perceptions in the classroom within our conceptual framework (Fig 1). The Assessment of Learner-Centered Practices (ALCP) [59], a faculty instrument, assessed characteristics of effective teaching, assessment of classroom practices most relative to motivation and achievement, and beliefs and assumptions about learners, learning, and teaching. The ALCP has been extensively validated and has undergone multiple item reliability analyses (α = 0.76–0.91) [60–62]. Two of the three scales within the ALCP measured learner-centered beliefs (*LC Bel*) and non-learner-centered beliefs (*NLC Bel*) of faculty. We expected learner-centered beliefs to fall closer to the learner-centered end of the gradient, while non-learner-centered beliefs may fall toward the teacher-centered end of the gradient (Fig 1). The Approaches to Teaching Inventory (ATI) [63], founded on research perspectives applied by Marton et al. [64], functioned to capture faculty approaches to teaching and learning; the ATI measured information-transfer/teacher-focused (*ITTF*) and conceptual change/student-focused (*CCSF*) practices. Prior studies have conducted psychometric analyses, including confirmatory factor analysis, on the ATI to ensure both its validity and reliability across a range of participants and settings (α = 0.66–0.74) [63, 65–66]. ITTF practices were expected to overlap with non-learner-centered beliefs at the teacher-centered end of the gradient, while CCSF practices were expected to overlap with learner-centered beliefs near the learner-centered end of the gradient (Fig 1).

**Table 1:** Five instruments for comparing perceptions of learner-centeredness. Within each student and instructor instrument exists primary and secondary subscales that we used in our study; we indicate the possible score ranges for each subscale and at which end of the learner-centered (LC) gradient a high score on that subscale would capture.

Focus Group	Tool	Primary Subscales	Secondary Subscales	Score range	High score captures which end of the LC gradient?	Citation
Instructor	ALCP	Non-learner-centered beliefs (NLC Bel)	*NLC-Bel*	5–20	Teacher-centered	[59]
		Learner-centered beliefs (LC Bel)	*LC-Bel*	5–20	Learner-centered	
Instructor	ATI	Info transfer/teacher-focused (ITTF)	*information transfer*, *teacher-focused*	8–40	Teacher-centered	[63]
		Conceptual change/student-focused (CCSF)	*conceptual change*, *student-focused*	8–40	Learner-centered	
Student	R-SPQ-2F	Deep approaches (Deep)	*deep motive*, *deep strategy*	10–50	Learner-centered	[43]
		Surface approaches (Surface)	*surface motive*, *surface strategy*	10–50	Teacher-centered	
Student	SETLQ	Knowledge & Learning Acquired (KLA)	Knowledge & subject-specific skills (*k-skills*), generic skills (*g-skills*), information skills (*i-skills*)	8–40	Learner-centered	[69]
		Experiences in Teaching & Learning (ETL)	*aims*, *choice*, *understanding*, *feedback*, *assessment*, *staff*, *students*, *interest*	25–125	Learner-centered	
Expert	RTOP	N/A	N/A	0–100	Learner-centered	[54]

The ALCP only contained primary subscales (NLC Bel and LC Bel), though these factors also served as a proxy for secondary subscale comparisons during our analyses across instruments. Additionally, the RTOP resulted in one average score per class session and we did not further break it down into primary or secondary subscales.

Two student surveys were used to evaluate student learning approaches on a deep or surface level and to better understand the general learning-teaching environment, respectively. The Revised 2-Factor Study Process Questionnaire (R-SPQ-2F) [43], based on the original Study Process Questionnaire (SPQ) developed by John Biggs in the 1980s, measured *deep* and *surface* approaches. Psychometric analyses including confirmatory factor analysis have been conducted by many prior researchers (α = 0.64–0.73) that suggest the R-SPQ-2F collects reliable data [43, 66–68]. While deeper approaches are motivated by a student’s intrinsic interests and desire to maximize meaning, surface approaches are motivated by a student’s fear of failure and rote learning strategies [43]. We expected deeper approaches to correspond with the learner-centered end of the gradient, while more surface approaches may fall on the teacher-centered end of the gradient (Fig 1). The Shortened Experiences of Teaching and Learning Questionnaire (SETLQ) [69] was produced as part of the Enhancing Teaching-Learning Environments in Undergraduate Courses Project and was intended to enhance student achievement via the strengthening of student-instructor relations and of the learning-teaching environment in general [69]. The SETLQ measured six scales, and we focused on two of those scales: student self-reported experiences of teaching and learning (*ETL*) and knowledge and learning acquired (*KLA*). Validity and reliability analyses for the SETLQ have been conducted in several prior studies (α = 0.56–0.83) [69–71].

We anticipated that students who self-reported increased learning gains in the classroom (KLA), in addition to having positive teaching and learning experiences (ETL), would cluster near the learner-centered end of the gradient; it should be noted that this is the only pair of subscales from a single instrument that were expected to associate with the *same end* (i.e. the learner-centered end) of the learner- and teacher-centered spectrum.

The Reformed Teaching Observation Protocol (*RTOP*) [54] quantified the learner-centeredness of instruction within each classroom, as determined by an external observer. The RTOP, originally designed by the Evaluation Facilitation Group of the Arizona Collaborative for Excellence in the Preparation of Teachers (ACEPT), allowed trained experts to objectively classify teaching in a classroom on the same learner- to teacher-centered spectrum described above (Fig 1). More learner-centered classrooms should earn higher RTOP scores, while more teacher-centered classrooms should earn lower RTOP scores. Sawada et al. [54] used RTOP to quantify the learner-centeredness of undergraduate science classrooms after instructors participated in professional development workshops.

In the current study, we chose to use RTOP rather than other expert observer tools such as the Classroom Observation Protocol for Undergraduate STEM (COPUS). RTOP requires more rigorous multi-day training to achieve sufficient interrater reliability [54], and contains protocol items that are more aligned with quantification of learner-centeredness in the classroom. Considering expert observer tools, RTOP was the best fit for our research objectives centered on learner-centeredness in the undergraduate biology classroom; per Sawada et al. [54], RTOP is “standards based, inquiry oriented, and student centered” (p. 1).

#### Administration and analysis of faculty instruments

Faculty surveys were administered online during the last week of the semester (via www.surveymonkey.com); however, instructors were given up to two weeks to complete the two faculty surveys to maximize response rates. In this study, ALCP [59] items were ranked on a 4-level Likert scale and ultimately, answers were categorized into either “learner-centered beliefs” or “non-learner-centered beliefs” (Scales 1 and 3, respectively); scores were then summed based on the system described by McCombs and Miller [59]. The ALCP Scale 2, or “Non Learner-Centered Beliefs about Learners,” was not used in this study, because it focused on personal reflection and emotional aspects of teaching [59, 72]. We felt that personal beliefs about student performance or persistence may or may not translate into an instructor’s pedagogical practices, thus did not cleanly overlay with one end of our framework, as we have defined it. The learner-centered beliefs and non-learner-centered beliefs subscales of the ALCP were not further broken down into secondary subscales as the other instructor and student instruments were.

The ATI consisted of sixteen five-point Likert scale items. Answers were ultimately characterized into one of two pedagogical categories of eight items each based on reported teaching practices: teacher-focused and information transfer-based *or* student-focused and conceptual change-based [63]. We then summed scores for items in each category. Within the ATI, ITTF can be further broken down into *information transfer* and *teacher-focused* and CCSF can be further broken down into *conceptual change* and *student-focused*. Hence, an instructor with a high ITTF score would tend to lecture at students more, while an instructor with a high CCSF score would generally focus more on students’ understanding of concepts rather than simply transferring knowledge.

#### Administration and analysis of student instruments

The R-SPQ-2F asked students to respond to twenty items related to attitudes towards and usual methods of studying; the scale for each item ranged from 1 (never or only rarely) to 5 (always or almost always). Main scale scores were categorized into one of two categories and summed: deep or surface approaches [43]. Within the R-SPQ-2F, the deep subscale can be further broken down into *deep motive* and *deep strategy*, while the surface subscale can be similarly broken down into *surface motive* and *surface strategy*. In this case, motive refers to a student’s justification for learning and succeeding in the classroom, while strategy refers to a student’s plan for learning the material in a particular course and how effective they are in doing so.

Although the SETLQ is composed of six sections, we used only two subscales (the ETL and KLA, described above) in this study due to our perception of their direct relevance to learner-centeredness. The ETL asked students to indicate their level of agreement on 25 items, of a 5-level Likert scale, based on their general approaches to studying and learning. The KLA asked students to respond to eight items regarding their perceptions of what they had learned in the course (i.e., Introductory Biology); the scale for each item ranged from 1 (very little) to 5 (a lot). Scores for each subscale were calculated by summing item responses in a given subscale. Within the SETLQ, the ETL can be further broken down into *Aims and congruence (aims)*, *Choice allowed (choice)*, *Teaching for understanding (understanding)*, *Set work and feedback* (*feedback)*, *Assessing understanding (assessment)*, *Staff enthusiasm and support (staff)*, *Student support (students)*, and *Interest and enjoyment (interest)*, while the KLA can be further broken down into *knowledge and subject-specific skills (k-skills)*, *generic skills (g-skills)*, and *information skills (i-skills)*.

Both student surveys were administered online during the last week of the semester (via www.surveymonkey.com) and students were given a week and compensated 1% of their final grade to complete them. Additionally, at the beginning of the semester, students were administered a demographic questionnaire and a critical thinking survey used for another study [11]. The demographic survey included seven questions and collected the ethnic and educational backgrounds of the student participants. Demographic information was available for 94% of students in the current study.

#### Collection and scoring of expert instrument

During Fall of 2013 and Spring of 2014, 65 classroom sessions of the 12 introductory biology sections were recorded. Filming days were generally selected at random, and each section was recorded between four to eight times during semester, usually without advance notice to the instructor. Three to four usable videos from each section were randomly selected to evaluate using the RTOP. We expected that analyzing multiple class sessions would provide a more comprehensive range of pedagogical strategies the instructors employed throughout the semester, hence representing a more genuine measure of learner-centeredness in the classroom. The RTOP is a tool, considered both valid [54, 73] and reliable [74–75], which quantitatively measures the learner-centeredness of instruction in a classroom. In this study, videos were independently rated by at least two trained raters and inter-reliability was high (generalizability coefficient = 0.787; see [11]).

Three scales exist within the RTOP, including lesson design and implementation, content, and class culture; items within each scale (25 total) were ranked on a scale from zero (absent) to four (present) [54]. The summed scores from the 25 items results in an RTOP lesson score ranging from 1–100. Two trained raters [11] independently scored each class session. Each score was categorized into one of five RTOP levels [37, 76]. If both raters’ scores categorized the same class session into the same RTOP level, the scores were averaged; however, if two scores for a single class session fell into different RTOP levels then an additional tie-breaker rater was used and the two scores sharing an RTOP level were used and averaged. Multiple class session RTOP scores for each section were averaged into a single score. We could not use the natural scales within RTOP, since our final RTOP score for each section represented an average among several raters and class sessions.

### Data and analyses

Cronbach’s reliability analyses for each scale were calculated in SPSS [77]. From the nine subscales representing three perspectives (student, instructor, and expert observer), we created five data matrices which were used in multivariate analyses. We initially created two sets of these five data matrices; one set used section (n = 12) as the sample unit and the other set used individual students (n = 490) as the sample unit. For each set, the first two matrices included student data: student primary subscales (4 factors) and student secondary subscales (15 factors). The next two matrices included faculty data: instructor primary subscales (4 factors) and instructor secondary subscales (6 factors). The final data matrix, RTOP scores (1 factor), represented expert observations of the same classes.

Pairwise Pearson correlations of univariate factors were run in SPSS [77]. We compared all our factors, including RTOP (expert) scores and student and faculty instruments, at either the primary subscale (i.e. ITTF, CCSF, LC-bel, NLC-bel, Deep, Surface, ETL, and KLA; Table 2) or secondary subscale (listed in italics in the *Administration and Analysis of Student/Faculty Instruments* sections above; S1 Table). Correlations were compared to a null hypothesis of no relationship, and the resulting p-values were compared to a Bonferroni-adjusted alpha of 0.000806 for the primary subscale comparisons (Table 2) and 0.000113 for the secondary subscale comparisons (S1 Table). The Bonferroni-adjusted alpha corrected for multiple comparisons to reduce the possibility of measuring false-positive results.

**Table 2:** Pearson correlations between primary instructor subscales, primary student subscales, and RTOP scores across all sections.

* *	* *	Instructor (ATI)		Instructor (ALCP)		Expert	Student (R-SPQ-2F)		Student (SETLQ)	
* *	* *	ITTF	CCSF	LC-bel	NLC-bel	RTOP	Deep	Surface	ETL	KLA
**Instructor (ATI)**	**ITTF**	1	-0.55	-0.54	0.19	-0.57	-0.16	0.15	-0.17	-0.45
	**CCSF**		1	0.36	-0.15	0.57	0.11	-0.74	0.81	0.77
**Instructor (ALCP)**	**LC-bel**			1	-0.23	0.32	0.18	0.20	-0.16	0.40
	**NLC-bel**				1	-0.28	-0.02	0.07	0.00	0.26
**Expert**	**RTOP**					1	0.60	-0.23	0.26	0.50
**Student (R-SPQ-2F)**	**Deep**						1	0.23	-0.18	0.28
	**Surface**							1	*-0.97	-0.37
**Student (SETLQ)**	**ETL**								1	0.53
	**KLA**									1

(*) indicates a significant relationship at the corrected alpha of 0.000806, compared to a null hypothesis of no relationship.

We ran nonmetric multidimensional scaling (NMS) analyses, using a Euclidean distance measure, in PC-ORD 7 [78] to identify gradients in perceptions of learner-centeredness and visually capture how various perceptions overlap. NMS is a multivariate ordination technique that represents the sample units in as few dimensions as possible using measured similarities. We chose NMS over other ordination techniques (e.g., PCA, factor analysis), because NMS allows you to select your own distance measure, and allows you to rigidly rotate your final configuration to align with a variable of interest rather than loading the greatest variance on the first axis. The purpose of our NMS analysis was to (1) test the hypothesis of our framework (i.e., if learner-centeredness is one-dimensional), (2) understand the relationship of secondary subscales to the learner-centered structure defined by primary scales, and (3) understand the relationship of faculty scales to the learner-centered structure defined by student scales [79–80].

We chose to use the student primary subscale data as the main matrix upon which to build ordinations and all other data as secondary matrices to investigate after-the-fact relationships with this matrix. We selected the student matrix, instead of the faculty matrix, because it represented a larger sample (i.e., 490 students vs. 7 faculty members); further, students are the natural center point of a learner-centered classroom, so we wanted to align all other perspectives to theirs.

Mantel tests differ from simple univariate correlations in that they measure correlations across matrices rather than individual pairwise comparisons. Our Mantel tests evaluated collective differences between students, instructors, and expert observers using all instruments together [81–82]. Lastly, cluster analyses using Ward’s minimum variance method [83] to estimate the expected number of clusters (based on a Euclidean distance measure) were run in PC-ORD 7 to further analyze how alike course sections were based on instructor versus student perceptions. Cluster analysis is a multivariate classification technique that separates data into meaningful groups (or clusters) based on overall relatedness; hence, items that cluster together are more related than items that do not cluster into the same group [84]. We used cluster analysis to independently separate primary student and instructor data into meaningful groups based on course section for later comparison.

### Data adjustments

Unfortunately, we found cluster analyses with student as the sample unit were unwieldly in size (i.e., 490 branch tips), not informative, and did not produce identifiable patterns within the cluster dendrograms. Further, the overall patterns in the ordinations and proportion of variance explained was similar using students or sections (i.e., all students within a section averaged) as sample units. We further discovered that secondary subscales in ordination analyses may be more accurate in parsing out perceptions of learner-centeredness with section as sample unit compared to using student responses as sample unit, though we found no difference in comparing primary subscales using section versus student responses as sample units. Particularly in science education, the use of individual student responses as sample units often leads to an inability to distinguish between learning gains due to instructional practices or learning gains due to extrinsic factors (e.g. experiences and backgrounds) of individual students [85]. While individual student responses may seem more attractive as a sample unit, they act as pseudoreplicates; therefore, sections as sample units are statistically superior. Results using students as sample units, therefore, are not reported here and all subsequent analyses reflect sections.

## Results

### Participating students, instructors, and class sections

To better describe our student population, we collected self-reported demographic data from our participants. Of the 490 students in our sample who fully completed the demographic portions of the student surveys, 30.8% (151 students) were freshmen, 43.3% (212) were sophomores, 19.6% (96) were juniors, 5.1% (25) were seniors, and 1.2% (6) were post-baccalaureate. The mean self-reported grade-point average within this student population was 3.3 on a 0.0–4.0 scale, while the mean ACT score was 22.9. The majority of participants (79%; 389 students) were Caucasian; 9% (46) were Latina/o; and 12% (55) were other ethnicities. Students, on average, had taken 1.2 biology courses in high school and 0.2 biology courses at the college level. Summary data for each instrument are available in Table 3.

**Table 3:** Summary statistics for student, instructor, and expert observer instruments.

Population	Instrument	Subscale	Score Range	Actual Minimum	Actual Maximum	Mean	Reliability (α)
Student	R-SPQ-2F	Deep	10–50	10	50	28.7	0.842
Student	R-SPQ-2F	Surface	10–50	10	50	28.2	0.805
Student	SETLQ	ETL	25–125	25	125	82.3	0.960
Student	SETLQ	KLA	8–40	8	40	26.4	0.899
Instructor	ATI	ITTF	8–40	17	33	23.9	0.727
Instructor	ATI	CCSF	8–40	20	32	27.1	0.534
Instructor	ALCP	LC Beliefs	5–25	11	20	15.6	0.781
Instructor	ALCP	Non-LC Beliefs	5–25	9	16	12.6	0.381
Expert	RTOP	N/A	0–100	32.17	54.42	40.1	0.787*

Low reliability of the CCSF subscale is most certainly skewed by the incredibly low reliability of the SF portion of the subscale (α = 0.090) rather than the CC portion of the subscale (α = 0.634). Low overall scale reliability for instructor subscales within the ALCP could be attributed to the low instructor sample size (n = 7); the ALCP incorporates a more affective dimension of learner-centeredness compared to the ATI, though this dimension could not be adequately detected based on instructors’ self-reported responses.

(*) 0.787 for RTOP represents the generalizability coefficient, or inter-rater reliability.

### Pairwise univariate correlations

#### Primary subscales

Comparing primary subscales (e.g. ITTF, CCSF, LC-bel, NLC-bel, Deep, Surface, ETL, and KLA) and RTOP across sections via Pearson correlations (Table 2), the strongest negative correlation was measured between ETL and Surface (r = -0.97; p < 0.000806), which represent student subscales from different instruments. We found no strong positive correlations between primary subscales (p > 0.000806; Table 2) across sections.

#### Secondary subscales

Secondary subscales identified above in the Methods were also compared across sections via Pearson correlations (S1 Table). We identified no strong negative nor positive correlations between any secondary subscales (p > 0.000113; S1 Table) across sections.

### Multivariate trends among instruments

#### Ordinations

**Fig 2:**
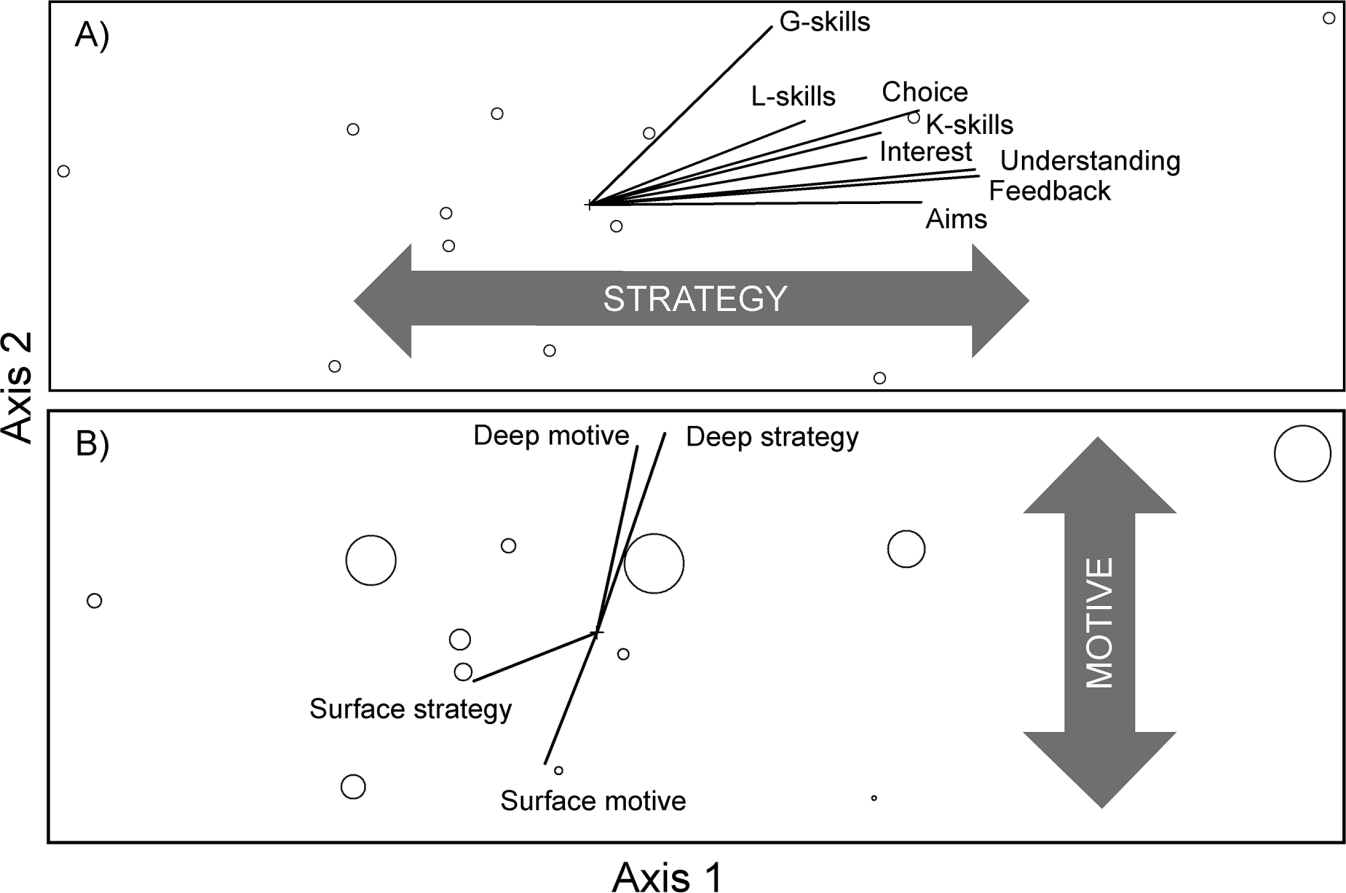
Twelve course sections are shown as open circles in student primary subscale space using NMS. (a) Several components of the ETL and KLA positively correlate with Axis 1, the strategy axis. *Conceptual change* of the ATI also correlated at the positive end of axis one, though was not included in the ordination figure. (b) The Deep and Surface approaches of the R-SPQ-2F associate with the positive and negative ends of Axis 2, the motive axis, respectively. In this panel, the relative symbol size of the 12 course sections are coded by RTOP score; high RTOP scores (i.e., larger circles) correlate with the positive end of Axis 2.

In analyzing average student responses of primary subscales (e.g. Deep, Surface, ETL, and KLA) across our twelve sections, the final stress for a two-dimensional solution was 1.2067 (p = 0.0199), with a final instability of <0.001 after 52 iterations (Fig 2). We rotated this ordination by the strongest variable, ETL (353 degrees), to load it on a single axis. Axis one explained 96.3% of the variance and axis two explained 3.3% of variance in student primary subscale scores. ETL (r = 0.99) and KLA (r = 0.83) explained most of the positive end of axis one, while the opposing end of axis one was associated with Surface approaches (r = -0.60). Axis two opposed Deep approaches (r = 0.91) and somewhat KLA scores (r = 0.57) at the positive end and Surface approaches (r = -0.67) at the negative end. The positive end of Axis 1 was characterized by learner-centered strategies, while the negative end was indicative of non-learner-centered strategies. Similarly, the positive end of Axis 2 was characterized by learner-centered motives, while the negative end was indicative of non-learner-centered motives (Fig 2).

When student secondary subscales by section were overlaid onto the student primary student subscales ordination, the positive end of axis one was associated with several of the secondary subscales, including those of the ETL (SETLQ): *feedback* (r = 0.97), *understanding* (r = 0.97), *choice* (r = 0.90), *aims* (r = 0.90), *interest* (r = 0.82), *staff* (r = 0.59), and *student* (r = 0.58); those of the KLA (SETLQ): *k-skills* (r = 0.84), *i-skills* (r = 0.72), and *g-skills* (r = 0.67); and one from the R-SPQ-2F: *deep strategy* (r = 0.53). *Assess* was the only secondary subscale of the ETL that did not strongly correlate with the positive end of axis one (r = 0.35). It should be noted that Deep approaches in the primary subscales above did not strongly associate with axis one, although strong correlations did arise among the Deep secondary subscales and axis one. The opposing end of axis one was only strongly associated with the R-SPQ-2F’s *surface strategy* (r = -0.72). The positive end of axis two was correlated with *deep strategy* (R-SPQ-2F; r = 0.91), *deep motive* (R-SPQ-2F; r = 0.88), and *g-skills* (r = 0.66), while *surface motive* (R-SPQ-2F; r = -0.74) was the only secondary subscale strongly related to the negative end of axis two (Fig 2).

When instructor primary subscales were overlaid onto the ordination of mean student responses per section in primary subscale space, CCSF (ATI) was related to the positive end of axis one (r = 0.63), while no factors were strongly associated (r > -0.5) with the negative end of axis one nor either end of axis two. When instructor secondary subscales were overlaid onto the student primary subscales, *conceptual change* (ATI) associated with the positive end of axis one (r = 0.61), while no factors were strongly associated (r > ±0.5) with the negative end of axis one nor either end of axis two. The single factor which captured expert perceptions, RTOP, correlated with the positive end of axis two (r = 0.68) but was not strongly associated with axis one. The primary subscales from the second instructor tool, the ALCP, were not strongly associated with either axis (r < ±0.5) (Fig 2).

### Multivariate correlations

Pairwise Mantel tests jointly compared multiple indices of student, instructor, and expert perceptions of the learner-centeredness of participating classes. No significant correlations (p < 0.05) existed among class sections based on similarities using primary subscales of instructors and students or RTOP (Table 4). Similarly, no significant correlations (p < 0.05) existed among class sections based on similarities using secondary subscales of instructors and students or RTOP (Table 4).

**Table 4:** Mantel tests between primary and secondary subscale scores. Correlation coefficients and p-values in upper corner compare primary subscale scores, while correlation coefficients in the lower corner compare secondary subscale scores.

* *	Instructor	Expert	Student
**Instructor**	1	p = 0.24; r = 0.16	p = 0.82; r = 0.03
**Expert**	p = 0.23; r = -0.16	1	p = 0.22, r = 0.20
**Student**	p = 0.13; r = 0.02	p = 0.20; r = 0.00	1

### Cluster analyses

**Fig 3:**
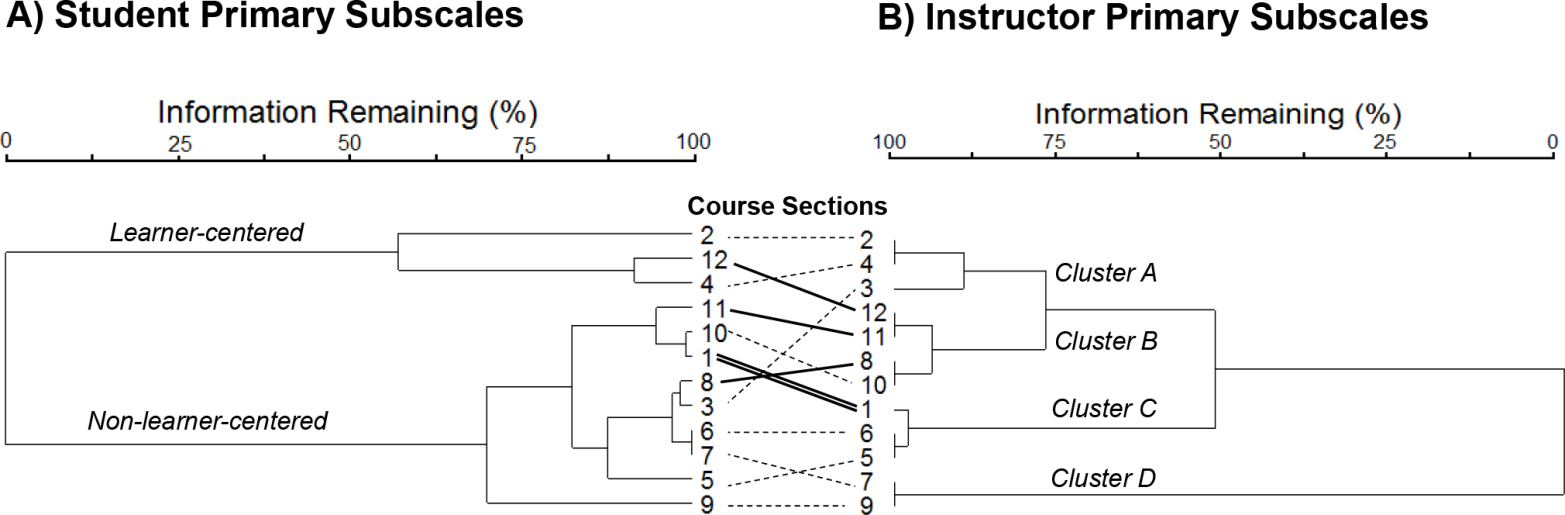
Twelve introductory biology course sections independently clustered by student and instructor primary subscales. In the dendrogram, information remaining (%) is indicative of the strength of the relationship between class sections; clusters joined with greater information remaining are more closely related. Sections are clustered by student perceptions in the dendrogram to the left (a), while the same sections are clustered by instructor perceptions in the right dendrogram (b). Identical course sections are connected in the center to aid in visualization of similarities; connector lines patterns denote enrollment size (dashed line ≤70 students, solid line = 71–150 students, bolded double line >150 students [one section, n = 391]). In the instructor dendrogram, Cluster A is the true learner-centered cluster; Cluster B is characterized by internal confusion within individual faculty; Cluster C is epitomized by the conflict in perspectives among groups; and Cluster D is the non-learner-centered cluster based on instructor and student perceptions.

To further analyze the relatedness of instructor to student perceptions of learner-centeredness, we compared independent cluster dendrograms based on section-averaged primary subscale responses. Dendrogram nodes were rotated to best align clusters of sections between student and instructor perspectives (Fig 3). Some pairs of course sections (i.e., 2 and 4; 11 and 12; 7 and 9; 5 and 6; and 8 and 10) were taught by the same instructor, thus their faculty survey scores are identical. In grouping course sections by student primary subscales (Fig 3A), we identified two main clusters with 50% information remaining. The first student cluster (top cluster; Fig 3A) included three course sections (i.e. 2, 12, and 4) in which students tended to have higher ETL, KLA, and deep scores and lower surface scores; this first group was categorized as the more learner-centered group in which learning was based on deep approaches. Interestingly, this cluster also included more of the low enrollment course sections (mean = 57.67 students per section, range = 48–75 students). The second student cluster (bottom cluster; Fig 3A) included nine course sections (i.e. 11, 10, 1, 8, 3, 6, 7, 5, and 9) in which students tended to have low ETL, KLA, and deep scores and high surface scores; this second group was categorized as the more non-learner-centered group in which learning was based on surface approaches. Interestingly, this cluster also appeared to include more of the higher enrollment course sections (mean = 102.67 students per section, range = 16–391 students).

In grouping course sections by instructor primary subscales (Fig 3B), we identified four main clusters with approximately 85% information remaining. The first faculty cluster (cluster A; Fig 3B) included three course sections (i.e. 2, 4, 3) in which instructors were more learner-centered as evidenced by high CCSF scores and three of the top four RTOP scores; interestingly, students also perceived two out of three of these moderately-sized classes to be learner-centered (Fig 3A). Cluster A is the only truly learner-centered cluster, where student, faculty, and expert perceptions of learner-centeredness tended to generally align.

The second faculty cluster, cluster B, included four course sections (i.e. 12, 11, 8, and 10) in which instructors were less learner-centered as evidenced by generally higher ITTF and NLC-bel scores; however, sections twelve and eleven had average to high CCSF and LC-bel scores while sections eight and ten had average CCSF and LC-bel scores (Fig 3B). The high CCSF scores in sections twelve and eleven are attributed to high *conceptual change* scores, as *student-focused* scores were quite low in these sections. Interestingly, the single instructor of these two sections had more than twenty years of teaching experience and earned relatively low RTOP scores. So while this instructor may have identified with the ideas of learner-centeredness in theory, they may not have put this theory into practice while teaching the sessions we observed. Notably, the instructor of sections 8 and 10 had little teaching experience, which likely influenced their counterintuitive perception of their own teaching as both teacher-focused and student-centered. Students within cluster B perceived these classes to be non-learner-centered, excepting for section 12, in which students perceived the class to be highly learner-centered (Fig 3A). Generally, students and experts agreed that the sections in cluster B were non-learner centered, while these instructors expressed mixed views of which end of the spectrum their teaching occupied. Three of the four sections in this second cluster had the greatest student enrollments, excepting section 10, which was closer to the average.

Faculty cluster C included three course sections (i.e. 1, 6, and 5), where instructors had low ITTF scores and high CCSF and LC-bel scores (Fig 3B). Cluster C epitomized the conflict in perspectives among groups; while these instructors ranked themselves as highly learner-centered, their students ranked all three of these course sections as non-learner-centered (Fig 3A), and experts rated section 1 as learner-centered yet the other two as transitioning to learner-centered. While section 1 had the largest enrollment (n = 391) and was taught during weekday mornings, sections 5 and 6 had the smallest enrollments (n = 16 and n = 30, respectively) and were taught at more non-traditional times (on weekday evenings and weekends, respectively).

Finally, faculty cluster D included two course sections (i.e. 7 and 9) in which the single instructor who taught both sections had high ITTF scores and low CCSF and LC-bel scores (Fig 3B); these two courses represented the most teacher-centered faculty cluster. Students agreed that these sections were non-learner-centered, and experts scored them as in the low range of the RTOP level 2, just above teacher-centered.

While most course sections within the instructor and student dendrograms could be roughly aligned (as denoted by straight or nearly straight dashed lines connecting Fig 3A and 3B), some misalignments of sections based on instructor primary subscales versus student primary subscales occurred. Expert scoring of the learner-centeredness of these sections, also did not necessarily agree with these designations. Additionally, student primary subscale scores of two sections taught by the same instructor were never more similar to one another than they were to scores from other instructors’ sections. For example, though sections 11 and 12 were taught by the same instructor, students perceived section 11 as non-learner-centered and section 12 as learner-centered.

## Discussion

### How did subscales within and among student instruments compare?

Most of the primary and secondary subscales of the SETLQ positively and linearly correlated, suggesting that students’ positive experiences with learning coincide with their perceived knowledge gained. Entwistle [86] reported similar associations linking classroom experiences with conceptual understanding and knowledge acquired, and noted that the extent of conceptual understanding or knowledge acquired may also be influenced by a student’s decision to approach learning at a deep or surface level. While students’ strategies and motives for learning were orthogonal in our analysis, Deep and Surface approaches fell at each opposing end of both ordination axes (Fig 2). The ETL, KLA, and *deep strategies* fell together at the learner-centered end of the same axis, axis one. This alignment supports the idea that students who report having more positive classroom experiences and highly valuing course content tend to adopt deeper strategies [87]. The alliance of the two student surveys administered in this study suggests that the R-SPQ-2F and SETLQ can be used in conjunction with one another to capture students’ strategies and motives, experiences in teaching and learning, and knowledge acquired on a learner- to non-learner-centered gradient.

### How did subscales within and among instructor instruments compare?

In univariate contrasts, neither primary nor secondary subscales of the ATI significantly related to one another, in agreement with prior studies [88]. Surprisingly, the two subscales of the ALCP did not significantly correlate to one another or any of the other faculty scales. Affective aspects of teaching, measured by the ALCP, were likely not captured with the other instruments we used in our study. Low reliability of ALCP scales within our sample population, particularly for the non-learner-centered beliefs subscale, suggests this tool is not reliable with our small instructor population (n = 7) and thus may be ineffective to measure our desired factor, learner-centeredness. The lack of alignment we observed between the ATI and ALCP, at least the learner-centered beliefs scale that was moderately reliable, might suggest there is an additional dimension of learner-centeredness among instructors that the ATI did not capture, and which may reflect affective rather than practical aspects of learner-centered pedagogy.

### Is learner-centeredness best represented as a one-dimensional gradient?

We found student perceptions of learner-centeredness in introductory biology classrooms are multidimensional (Fig 2). Most of the variance among class sections, however, is loaded along one gradient, in line with our original hypothesis that perceptions of learner-centeredness would fall on a single-dimensional framework with two opposing ends. In the student survey, the R-SPQ-2F, the two secondary subscale factors (i.e., strategy and motive) became important but separate factors with surface and deep ends, which defined our two ordination gradients. While strategy represents one’s process or plan for learning, and motive represents one’s orientation for learning, it is important to keep in mind that multiple motive-strategy combinations may be possible; for example, a student may have deep motives but surface strategies for learning a topic [89].

We defined Axis 1 as the strategy gradient. Positive experiences of teaching and learning, increased knowledge acquired, deep strategies, and conceptual change describe the learner-centered end of this axis, whereas surface approaches describe the opposing, teacher-centered end (Fig 2). While the various primary and secondary subscales measured in this study did not covary using linear, univariate analyses, many of the subscales did overlay when viewed in multidimensional space; all subscales on Axis 1 (i.e. KLA, ETL, *conceptual change*, and *deep strategies*) aligned as predicted (Fig 1). The fact that *LC-beliefs* did not correlate with these other learner-centered measures may suggest that the ALCP is capturing an additional dimension of learner-centeredness (e.g., perhaps one more focused on affective aspects of instruction). Further, though *conceptual change* and *student-focused* comprised the CCSF subscale of the ATI, *student-focused* did not align with other measures of learner-centeredness. Elsewhere, secondary science teachers who intended to teach toward conceptual change rather than based on information transfer often were not able to implement student-focused practices into their lessons [90], which might explain the disconnect we measured between *conceptual change* and *student-focused* of the CCSF in the current study. Moreover, we also cannot overlook the considerable unreliability of the SF subscale in our sample, which likely disrupted any potential underlying trend. Low reliability of the CCSF subscale was most certainly skewed by the incredibly low reliability of the SF portion of the subscale (α = 0.090) rather than the CC portion of the subscale (α = 0.634).

We labeled Axis 2 as the motive gradient. At one end of this gradient, students expressed deep motives and strategies for learning and increased general learning skills, and experts perceived these classrooms as highly learner-centered. Surface motives defined the opposing end of this gradient (Fig 2). Sambell, Brown, and McDowell [91] noted that even in a learner-centered environment, a student may not adopt deep learning strategies if he or she is not motivated to engage in high-quality learning. However, students in a classroom are reportedly more motivated to succeed if they perceive that they have some control of their learning [92]. Further, alignment of expert and student perceptions of learner-centeredness has also been reported previously, including the correlation of high RTOP scores with student conceptual gains and classroom collaboration in a learner-centered course [53].

In its entirety, Axis 1 (i.e. the strategy gradient) explained substantially more variance in student scores; thus, may be more informative of students’ perceptions of learner-centeredness than Axis 2 (i.e. the motive gradient). While many have discussed the close relationship between conceptions of learning and approaches to learning [43, 93], others have argued that the interplay between conceptions of learning, approaches to learning, and extraneous factors such as culture is more complicated than a simple causal relationship [94–95]. While the design of our study cannot infer causation, the strategies students use correlate with a perceived gain in learning (in the form of ETL and KLA scores), but motive is uncoupled from strategy. Though some prior studies have reported that students engaging in deep strategies may not always possess deep motives for learning in a particular course, and vice versa [89], other studies have discussed the strong coupling of deep intrinsic motives and strategies among undergraduate students [96]. Further, students may perceive their strategies and motives as quite separate entities in the learning process [89], which could be related to the idea that students’ conceptions of learning (e.g. motives) may influence their approaches to learning [97–98], whether deep or surface.

### Are two dimensions of learner-centeredness enough?

Instructor perceptions of learner-centeredness, as measured by the CCSF and CC secondary subscale of the ATI, agreed with student perceptions and fell along the strongest gradient of learner-centeredness, the strategy gradient (Fig 2). While instructors in our sample may desire learner-centered outcomes in their classes (i.e., high CC), some do not engage in the necessary pedagogy to ensure a learner-centered class (i.e., high SF). The paradox of conceptual change in the absence of student-focused learning has been discussed by others in the context of limitations of the original conceptual change model—mainly, that there was too much focus on the instructor’s role, rather than the student’s role, in facilitating conceptual change in the classroom [33, 99–101]. A class based largely on conceptual change is perceived by our sampled students as a class requiring deep strategies and promoting positive learning experiences and increased knowledge and learning. Interestingly, Trigwell et al. [55] found that student-focused instructors were more likely to encourage deep learning approaches, which our data did not support since high CCSF scores in the current study were mainly driven by the *conceptual change* secondary subscale rather than the *student-focused* one. The *student-focused* subscale was not strongly correlated (r <0.50) to either student gradient, which may suggest additional dimensionality was perceived by instructors but not by students.

Similarly, the two subscales of the ALCP and the ITTF scale of the ATI did not associate with either gradient that students identified as learner-centered. This lack of relationship between the ALCP and other subscales within this study lends more evidence for the multi-dimensional framework of learner-centeredness, even beyond the 2-D model identified in our student ordination (Fig 2), rather than the one-dimensional framework described by our null hypothesis. The ALCP, as an example, describes faculty affect that may represent its own separate dimension of learner-centeredness with no relation to the motive and strategy gradients we identified. While prior studies have found strong associations between affective traits of teachers and student outcomes [102], affective measures of instructors have not historically been linked to instructor and student perceptions of learner-centeredness, as was done in this study by using multiple tools to quantify perceptions of each group.

### How did subscales across student, faculty, and expert observer instruments compare?

All univariate and multivariate linear correlations showed no relationships among the student, faculty, and expert instruments, which suggests a disconnect across the subscales of these instruments. However, using data reduction and agglomeration techniques (i.e., ordination and cluster analysis), we were able to identify some overlap in learner-centered perceptions. We found that expert and faculty perceptions mostly align based on cluster analysis; that expert and student perceptions align along the motive axis of the ordination; and that student and faculty perceptions generally do not agree, with the exception of the *conceptual change* subscale correlating with the learner-centered strategy end of axis one within the ordination.

Similar to our original hypothesis, as guided by work from Ebert-May et al. [37], our univariate contrasts suggested that expert perceptions of learner-centeredness (i.e. RTOP scores) generally did not relate to faculty perceptions, though our cluster analyses suggested that instructors who perceived their practices and beliefs as learner-centered often taught course sections that were more learner-centered based on expert opinions. Additionally, RTOP scores only associated with the weaker of the two student ordination axes, suggesting that experts’ perceptions of the classroom learner-centeredness more closely aligned with students’ perceptions of motives rather than strategies. Finally, in agreement with previous work [57], student and faculty perceptions of learner-centeredness were disconnected in all analyses with one exception (i.e., CC subscale positively associating with the student strategy gradient). Our findings contradict the general agreement between student and instructor perceptions identified by Trigwell et al. [55] using several of the same instruments administered in the current study, though Trigwell and others noted the small sample size that included only one field of study (i.e., physical science) warranted caution in interpreting the results. Likewise, our study included a relatively small sample (n = 12 class sections) restricted to a single discipline (i.e., biology), which may also contribute to the lack of agreement between our work and Trigwell and others [55].

Instructors in our study appear to perceive additional dimensions of learner-centeredness that students do not (i.e., measured by the subscales of ALCP), perhaps dimensions based more on affective aspects of teaching and learning. Sutton and Wheatley [103] discuss the emotional process as relevant to teaching, including how emotional expression and subjective tendencies of teachers may vary during instruction. The ALCP may incorporate this more affective dimension of learner-centeredness, though this dimension could not be adequately detected or aligned with other factors in the current study.

Our finding that RTOP did not associate with the strategy axis of the ordination (i.e., Axis 1) suggests that student strategies do not relate to observable classroom environment and behaviors. As mentioned above, students engaging in deep strategies may not always possess deep motives for learning in a particular course [89]. Perhaps the deep motives that many students fostered in the current study were influenced by positive aspects of the classroom environment such as group discussions with peers and a supportive instructor [104], though these motives may not have necessarily reflected students’ strategies to learn biology.

### Are perceptions of learner-centeredness biased by external factors?

In our sample, we found that the combination of low enrollment courses (i.e., less than or equal to 70 students) with high RTOP scores (i.e., greater than 40) could be viewed as highly learner-centered by both students and faculty. However, in classes where experts and faculty aligned as highly learner-centered yet were either very high enrollment (i.e., greater than 150 students) or taught during non-traditional times (evenings or weekends), students rated these sections as teacher-centered. Differential student success has elsewhere been tied to course scheduling; specifically, students in morning classes outperform students in non-morning classes [105]. Likewise, college science instructors often anecdotally feel that class size is a limitation in implementing more learner-centered or inquiry-based techniques in the lecture [106]. Our data empirically suggest that even if a class looks and feels learner-centered, external barriers (i.e., time of day, class size) may limit this perception by students.

Prior studies have concluded that learner-centered practices can be implemented effectively in large enrollment science courses [12, 33, 107]. However, our findings demonstrate that while faculty and experts perceive some larger enrollment course sections as learner-centered, students fail to perceive this learner-centeredness when enrolled in these large classes themselves. The tendency of students to perceive larger classes as more teacher-centered in the current study is similar to the trend described by Ebert May et al. [37] and Murray and MacDonald [108], though in these prior studies, instructors and experts, rather than students, perceived larger classes as more teacher-centered.

### Limitations

Though we assumed that the fifteen class sections and nine instructors in our study were representative of average undergraduate biology classrooms, our findings should be generalized with caution. We conducted our study at a single institution with nine instructors that collectively taught fifteen sections of the same non-majors introductory biology course. Expanding to include other institutions, science and non-science courses, and a variety of instructors could provide more generalizable patterns. Further, our study was conducted exclusively in biology courses, though none of the student, instructor, or expert instruments used in our study included items specific to the biological sciences; findings may be different if this research was conducted in other disciplines both within and beyond the sciences.

Future work could conduct additional psychometric analyses, especially on instruments or scales we found to be unreliable, or conduct qualitative interviews to provide additional validity for each instrument administered. The absence of clear patterns in some of our data analyses may reflect issues with the instruments themselves rather than a real trend, or lack thereof. Future directions of this research should also consider interventions to better align perceptions of learner-centeredness in the biology classroom, specifically focused on large or non-traditionally timed courses.

## Conclusions

Our sample of introductory biology classrooms clearly implies that learner-centeredness is multidimensional, as seen in our ordination, and is more complex than a simple dichotomous learner- versus teacher-centered relationship. The alignment of student, instructor, and expert perceptions of learner-centeredness or teacher-centeredness was generally inconsistent across sections of this non-majors biology course. While pairwise univariate correlations suggest few significant relationships exist between individual scales, Mantel tests indicate that perspectives measured by several instruments do not significantly correlate across students, instructors, and experts. Alternatively, our ordination highlights that student and expert perceptions of learner-centeredness align based on motives. Lastly, cluster analyses separated student and instructor data into meaningful groups, which suggest alignment of faculty and expert perceptions occurs in most contexts. Broadly, expert opinions tended to agree with instructor and student perceptions independently, while students’ perceptions mostly differed from those of faculty. Regretfully, the classroom experience for students can be negatively influenced by external factors, including enrollment size and time of lecture. Perceptions of learner-centeredness in the biology classroom are complex, and can be more completely measured and interpreted with more than one instrument. Our findings encourage instructors to be cognizant that the approaches they employ in the classroom may not be interpreted as learner-centered, in the same manner, by students and external observers, particularly when additional course factors such as enrollment and scheduling may encourage negative perceptions of learner-centered practices.

## Supporting information

S1 Table Pearson correlations between secondary instructor and student subscales and RTOP across all sections. (DOCX)
