# Supplementary material for: Comparing student, instructor, and expert perceptions of learner-centeredness in post-secondary biology classrooms
Source: PLoS One. 2018 Jul 11;13(7):e0200524. doi: 10.1371/journal.pone.0200524 (PMC6040760; doi:10.1371/journal.pone.0200524)
Supplement: S1 Table — (DOCX) [file pone.0200524.s001.docx]

|  |  | **Instructor (ATI)** | | | | **Instructor (ALCP)** | | **Expert** | **Student (R-SPQ-2F)** | | | | **Student (SETLQ)** | | | | | | | | | | |
| --- | --- | --- | --- | --- | --- | --- | --- | --- | --- | --- | --- | --- | --- | --- | --- | --- | --- | --- | --- | --- | --- | --- | --- |
|  |  | *Teacher-focused* | *Info transfer* | *Student-focused* | *Conceptual change* | *LC-bel* | *NLC-bel* | *RTOP* | *Deep motive* | *Deep strategy* | *Surface motive* | *Surface strategy* | *Aims* | *Choice* | *Understanding* | *Feedback* | *Assessment* | *Staff* | *Student* | *Interest* | *K-skills* | *G-skills* | *I-skills* |
| **Instructor (ATI)** | *Teacher-focused* | 1.00 | 0.67 | -0.80 | -0.36 | -0.67 | -0.08 | -0.28 | -0.05 | -0.31 | -0.04 | 0.33 | -0.18 | -0.43 | -0.29 | -0.43 | 0.13 | 0.11 | -0.56 | -0.42 | -0.17 | -0.40 | -0.68 |
|  | *Information transfer* |  | 1.00 | -0.52 | -0.37 | -0.44 | 0.54 | -0.54 | 0.10 | -0.19 | -0.16 | 0.11 | 0.24 | 0.14 | 0.18 | -0.01 | 0.09 | 0.12 | -0.44 | -0.08 | 0.17 | -0.17 | -0.18 |
|  | *Student-focused* |  |  | 1.00 | 0.34 | 0.71 | -0.05 | 0.47 | 0.47 | 0.70 | -0.14 | -0.31 | 0.15 | 0.50 | 0.31 | 0.46 | -0.16 | -0.15 | 0.89 | 0.54 | 0.22 | 0.68 | 0.56 |
|  | *Conceptual change* |  |  |  | 1.00 | 0.51 | -0.36 | 0.59 | -0.02 | 0.30 | -0.27 | -0.46 | 0.40 | 0.43 | 0.51 | 0.63 | 0.37 | 0.47 | 0.54 | 0.45 | 0.57 | 0.59 | 0.51 |
| **Instructor (ALCP)** | *LC-bel* |  |  |  |  | 1.00 | -0.23 | 0.32 | -0.01 | 0.29 | -0.11 | -0.17 | -0.02 | 0.26 | 0.12 | 0.33 | -0.22 | -0.28 | 0.61 | 0.34 | 0.14 | 0.56 | 0.46 |
|  | *NLC-bel* |  |  |  |  |  | 1.00 | -0.28 | 0.30 | 0.05 | -0.22 | -0.17 | 0.41 | 0.56 | 0.44 | 0.23 | -0.34 | -0.17 | -0.27 | 0.25 | 0.35 | -0.02 | 0.46 |
| **Expert** | *RTOP* |  |  |  |  |  |  | 1.00 | 0.55 | 0.70 | -0.52 | -0.46 | 0.09 | 0.36 | 0.29 | 0.38 | -0.18 | -0.05 | 0.63 | 0.51 | 0.38 | 0.56 | 0.40 |
| **Student (R-SPQ-2F)** | *Deep motive* |  |  |  |  |  |  |  | 1.00 | 0.88 | -0.62 | -0.38 | 0.21 | 0.57 | 0.41 | 0.34 | -0.26 | -0.16 | 0.54 | 0.49 | 0.33 | 0.50 | 0.32 |
|  | *Deep strategy* |  |  |  |  |  |  |  |  | 1.00 | -0.64 | -0.53 | 0.26 | 0.59 | 0.46 | 0.49 | -0.21 | -0.05 | 0.77 | 0.52 | 0.47 | 0.78 | 0.53 |
|  | *Surface motive* |  |  |  |  |  |  |  |  |  | 1.00 | 0.75 | -0.25 | -0.51 | -0.53 | -0.50 | 0.21 | 0.04 | -0.27 | -0.33 | -0.49 | -0.43 | -0.51 |
|  | *Surface strategy* |  |  |  |  |  |  |  |  |  |  | 1.00 | -0.50 | -0.63 | -0.71 | -0.76 | -0.17 | -0.34 | -0.41 | -0.55 | -0.58 | -0.43 | -0.70 |
| **Student (SETLQ)** | *Aims* |  |  |  |  |  |  |  |  |  |  |  | 1.00 | 0.81 | 0.91 | 0.83 | 0.32 | 0.64 | 0.25 | 0.68 | 0.85 | 0.51 | 0.61 |
|  | *Choice* |  |  |  |  |  |  |  |  |  |  |  |  | 1.00 | 0.93 | 0.87 | 0.00 | 0.25 | 0.51 | 0.82 | 0.82 | 0.66 | 0.84 |
|  | *Understanding* |  |  |  |  |  |  |  |  |  |  |  |  |  | 1.00 | 0.95 | 0.25 | 0.51 | 0.43 | 0.78 | 0.84 | 0.56 | 0.76 |
|  | *Feedback* |  |  |  |  |  |  |  |  |  |  |  |  |  |  | 1.00 | 0.28 | 0.49 | 0.60 | 0.85 | 0.78 | 0.62 | 0.77 |
|  | *Assessment* |  |  |  |  |  |  |  |  |  |  |  |  |  |  |  | 1.00 | 0.89 | 0.10 | 0.04 | 0.07 | -0.10 | -0.18 |
|  | *Staff* |  |  |  |  |  |  |  |  |  |  |  |  |  |  |  |  | 1.00 | 0.11 | 0.22 | 0.45 | 0.15 | 0.09 |
|  | *Student* |  |  |  |  |  |  |  |  |  |  |  |  |  |  |  |  |  | 1.00 | 0.67 | 0.32 | 0.73 | 0.44 |
|  | *Interest* |  |  |  |  |  |  |  |  |  |  |  |  |  |  |  |  |  |  | 1.00 | 0.65 | 0.57 | 0.62 |
|  | *K-skills* |  |  |  |  |  |  |  |  |  |  |  |  |  |  |  |  |  |  |  | 1.00 | 0.74 | 0.76 |
|  | *G-skills* |  |  |  |  |  |  |  |  |  |  |  |  |  |  |  |  |  |  |  |  | 1.00 | 0.66 |
|  | *I-skills* |  |  |  |  |  |  |  |  |  |  |  |  |  |  |  |  |  |  |  |  |  | 1.00 |
